# Dynamics of Daily Ageism and Attitudes for Healthy Aging: Cross-Cultural Analysis of the Subjective AGES Project

**DOI:** 10.1093/geroni/igaf122.1843

**Published:** 2025-12-31

**Authors:** Shevaun Neupert, Reyyan Can

## Abstract

The proposed symposium provides a global perspective regarding antecedents, correlates, and consequences of views on aging based on the Subjective AGES (Aging within Global everyday Ecological Studies) consortium. This culture-informed approach highlights the contextual and dynamic influences of daily ageism, attitudes, and behaviors across different temporal perspectives. We enrich the existing body of knowledge by including a broad variety of cultures and investigating how daily ageism and attitudes connect to daily indicators of health and well-being. First, Neupert et al. characterize the amount of day-to-day fluctuation in daily ageist attitudes across daily diary studies from 10 countries and show cross-cultural differences in the impact of those fluctuations on daily memory functioning. Wirth et al. use daily diary data from Germany, Israel, Türkiye, and USA and find that people with more age-related losses endorse more ageist attitudes than those with fewer losses. Lee et al. examine three exposure contexts as potential sources of daily ageist attitudes in Canada, finding that greater overall social media use, but not TV viewing or neighborhood age composition, was significantly associated with greater experiences of ageism in daily life. Röcke et al. connect fluctuations in subjective age with mobility in Switzerland, showing that participants who report higher flexibility concerning environmental and personal challenges travel further away from home on days when feeling younger than their actual age. Our findings shed light on country-specific and global relationships between views on aging and development. We showcase how experiences of aging are shaped by contextual and dynamic influences.

